# Analysis of clinicopathological characteristics and prognostic factors of early‐stage human epidermal growth factor receptor 2 (HER2)‐low breast cancer: Compared with HER2‐0 breast cancer

**DOI:** 10.1002/cam4.6571

**Published:** 2023-09-29

**Authors:** Qian Wu, Fan Yang, YinHua Liu, Hong Zhang, Shuang Zhang, Ling Xin, Ling Xu

**Affiliations:** ^1^ Thyroid and Breast Surgery Peking University First Hospital Beijing China; ^2^ Department of Hepatobiliary and Pancreatic Surgery The First Affiliated Hospital, Zhejiang University School of Medicine Hangzhou China; ^3^ Department of Pathology Peking University First Hospital Beijing China; ^4^ Department of Pathology National Cancer Center/National Clinical Research Center for Cancer/Cancer Hospital, Chinese Academy of Medical Sciences and Peking Union Medical College Beijing China

**Keywords:** breast cancer, early stage, HER2‐low, prognosis

## Abstract

**Purpose:**

To investigate the clinicopathological characteristics and prognostic factors of early‐stage breast cancer (EBC) with human epidermal growth factor receptor 2 (HER2)‐low expression.

**Methods:**

The clinicopathological data and follow‐up information of EBC patients with HER2‐low and HER2‐0 expression treated at the Breast Disease Center of Peking University First Hospital from January 2014 to December 2017 were analyzed. The prognosis between HER2‐low and HER2‐0 expression groups and with different hormone receptor (HR) expression were compared by statistics. Meanwhile, the expression of Ki67, androgen receptor (AR), TOPIIa, P53, PTEN, and CK5/6 were also analyzed with the HER2‐low expression and prognosis.

**Results:**

Retrospectively analyzed 1253 cases of EBC, including 583 (46.5%) cases of HER2‐low breast cancer (BC) and 366 (29.2%) HER2‐0 BC cases. Among the HER2‐low BC patients, 487 (83.5%) were HR‐positive, while 96 (16.5%) were HR‐negative. Among the HER2‐0 BC patients, 265 (72.4%) were HR‐positive, while 101 (27.6%) were HR‐negative. Median follow‐up time was 53 months. The 5‐year disease‐free survival of HER2‐low BC patients was 90.2% (95% confidence interval [CI]: 87.2–93.1), and the 5‐year overall survival was 95.4% (95% CI: 93.3–97.6). Cox regression analysis showed that T stage, lymphovascular invasion, and/or perineural invasion were prognostic factors of HER2‐low BC patients. However, the 5‐year disease‐free survival and overall survival of patients in the HER2‐low and HER2‐0 groups were not significantly different in all patients, but a tendency of better prognosis in HER2‐low group was seen in HR‐negative tumors.

**Conclusion:**

HER2‐low EBC patients accounted for 46.5% of the patient population. T stage, lymphovascular invasion, and/or perineural invasion were factors affecting the prognosis of BC patients with low HER2 expression. No significant difference in prognosis was noted between HER2‐low and HER2‐0 EBC patients. But in HR‐negative tumors, a tendency of better prognosis was seen in HER2‐low versus HER2‐0.

## 1 INTRODUCTION

Since the beginning of the 21st century, breast cancer (BC) has gradually entered the era of individualized treatment. Targeted drugs such as trastuzumab and pertuzumab have provided significant survival benefits to human epidermal growth factor receptor 2 (HER2)‐positive BC patients.^1^ In 2021, with high‐level evidence, BC clinical practice guidelines^2^, ^3^ continued to recommend trastuzumab or trastuzumab + pertuzumab combination chemotherapy regimens as neoadjuvant/adjuvant therapy for HER2‐positive BC patients with indications. The successful development of an antibody–drug conjugate (ADC) targeting HER2 and the findings of its survival benefit^4^ have led some to consider refining HER2 expression status and exploring factors related to the prognosis of HER2‐low BC patients. To analyze the clinicopathological characteristics and prognostic factors of HER2‐low BC patients, we collected and analyzed the clinicopathological data of HER2‐low early‐stage breast cancer (EBC) patients treated at the Breast Disease Center of Peking University First Hospital from January 2014 to December 2017. We present the following article in accordance with the STROBE reporting checklist.

## 2 METHODS

### 2.1 Patients

The clinicopathological data of EBC patients from the database of the Breast Disease Center of Peking University First Hospital from January 1, 2014, to December 31, 2017, were retrospectively analyzed. The study was conducted in accordance with the Declaration of Helsinki (as revised in 2013). The study was approved by the Biomedical Research Ethics Committee of Peking University First Hospital (No. 2021 Research 206), and individual consent for this retrospective analysis was waived.

Inclusion criteria: female; invasive BC histopathologically confirmed by breast lesion core‐needle biopsy; EBC: early‐stage breast cancer with T1‐4, N0‐3, and M0 at diagnosis; HER2 immunohistochemistry (IHC) and in situ hybridization (ISH) performed according to the 2013 American Society of Clinical Oncology/College of American Pathologists (ASCO/CAP) guidelines and meeting the diagnostic criteria for HER2‐low (Method 2.1.2); a treatment plan standardized according to the BC clinical practice guidelines of the National Comprehensive Cancer Network (NCCN) or the BC diagnosis and treatment guidelines of the Chinese Society of Clinical Oncology (CSCO); R0 resection completed; and complete clinicopathology and follow‐up data.

Exclusion criteria: male; breast cancer with metastasis at diagnosis; multiple primary invasive breast cancer lesions with an inconsistent HER2 status; no standardized systemic treatment or surgical treatment (Method 2.4); unclear HER2 status; and incomplete clinicopathological data and follow‐up data. Due to different molecular expression and biological behavior between male and female breast cancer, male patients were not included in this study in order to ensure the consistency of the research results.

### 2.2 Clinical and histopathological evaluation

Standardized clinical and histopathological evaluation was performed in all patients.

The histopathological evaluation was performed according to the World Health Organization tumor classification and the standardized procedures of CAP for invasive breast cancer.^5^, ^6^ Items including the tumor T stage, N stage, pathological type, histological grade, lymphovascular invasion, and perineural invasion were included in the evaluation.

Evaluation of HER2 status was performed according to the 2013 ASCO/CAP criteria.^7^ The laboratory was certified by the National Pathology Quality Control Center (PQCC). The VENTANA 4B5 antibody was used for IHC detection and was performed on the VENTANA automated immunohistochemistry platform using a standardized procedure. The HER2 gene test kit (fluorescence ISH; Guangzhou Anbiping Medicine Technology Co., Ltd.) was used for ISH. These materials were all approved by the National Medical Products Administration (NMPA). The criteria for HER2 positivity were as follows: IHC 3+ (>10% of infiltrating cancer cells showed strong, intact, and uniform cell membrane staining); or IHC 2+ (>10% of infiltrating cancer cells showed weak‐moderate intact cell membrane staining or ≤10% of infiltrating cancer cells showed strong and intact cell membrane staining); and positive ISH (a HER2/CEP17 ratio ≥2.0 and an average HER2 copy number ≥4.0 signals/cell or a mean HER2 copy number ≥6.0 signals/cell). The criteria for HER2‐low were as follows: IHC 1+ (>10% of infiltrating cancer cells showed incomplete and weak cell membrane staining) or IHC2+ plus ISH negativity (a HER2/CEP17 ratio ≥2 and an average HER2 copy number <4.0 signals/cell or a HER2/CEP17 ratio <2.0 and an average HER2 copy number <6.0 signals/cell). The criteria for HER2‐0 were an IHC score of 0 (no staining or ≤10% of infiltrating cancer cells showed incomplete and weak cell membrane staining).

ER and PR were evaluated according to the 2010 ASCO/CAP criteria.^8^ Ki67 was evaluated according to the 2011 International Ki67 Breast Cancer Working Group Guidelines.^9^ Tumor‐infiltrating lymphocytes (TILs) were evaluated according to the 2014 International TILs Working Group Guidelines.^10^ Other biomarkers in the surgical specimens, including CK5/6, EGFR, PTEN, p53, AR, and TOPIIa, were also detected by IHC, whose representative images were shown in Figure S1.

Stage: Tumor (T)–lymph node (N)–metastasis (M) stage, anatomical stage, and prognostic stage were evaluated followed the American Joint Committee on Cancer (AJCC) BC staging system (8th edition).^11^

Surrogated Molecular type: Surrogated Molecular type was evaluated according to the 2013 St. Gallen International Expert Consensus by combining ER, PR, HER2, and Ki67.^12^ Basal‐like subtype was defined as ER negative, CK5/6, and (or) EGFR positive.^13^

### 2.3 Treatment regimens and follow‐up

Breast surgery included lumpectomy and total mastectomy. Axillary surgery included sentinel lymph node biopsy and axillary lymph node dissection. Axillary lymph node dissection covered the ipsilateral axillary lymph nodes at levels I and II.^14^ Systemic treatment was performed according to the NCCN guidelines or the CSCO guidelines, with adjuvant/neoadjuvant chemotherapy ≥4 cycles and adjuvant endocrine therapy ≥3 years.

The primary endpoint of this study was 5‐year disease‐free survival (DFS), which was measured from the date of treatment to the date of recurrence and metastasis. The secondary endpoint of the study was 5‐year overall survival (OS), which was measured from the date of BC diagnosis to the date of death from any cause. All patients were followed up every 6 months, and the last follow‐up date was December 2020. The follow‐up included ultrasound of the breast and axillary nodes, abdominal ultrasound/computed tomography (CT), chest X‐ray/chest CT, and other necessary examinations.

### 2.4 Statistical analysis

Measurement data are described using the median and interquartile range values; count and ranked data are described using the number of cases and percentages. To compare two groups, the Mann–Whitney test was used for continuous variables, and Pearson's χ^2^ test or Fisher's exact test was used for categorical variables. Prognostic analysis was performed using the Kaplan–Meier method to plot the survival curve, and the log‐rank test was used. Cox regression analysis was performed to perform univariate survival analysis of prognostic factors. Factors with *p* < 0.1 were included in the multivariate analysis. All tests were performed using a two‐sided test. *p* < 0.05 was considered statistically significant. All analyses were performed with R 3.6.3 and SPSS 23.0 (SPSS Inc.).

## 3 RESULTS

### 3.1 Patients

**FIGURE 1:**
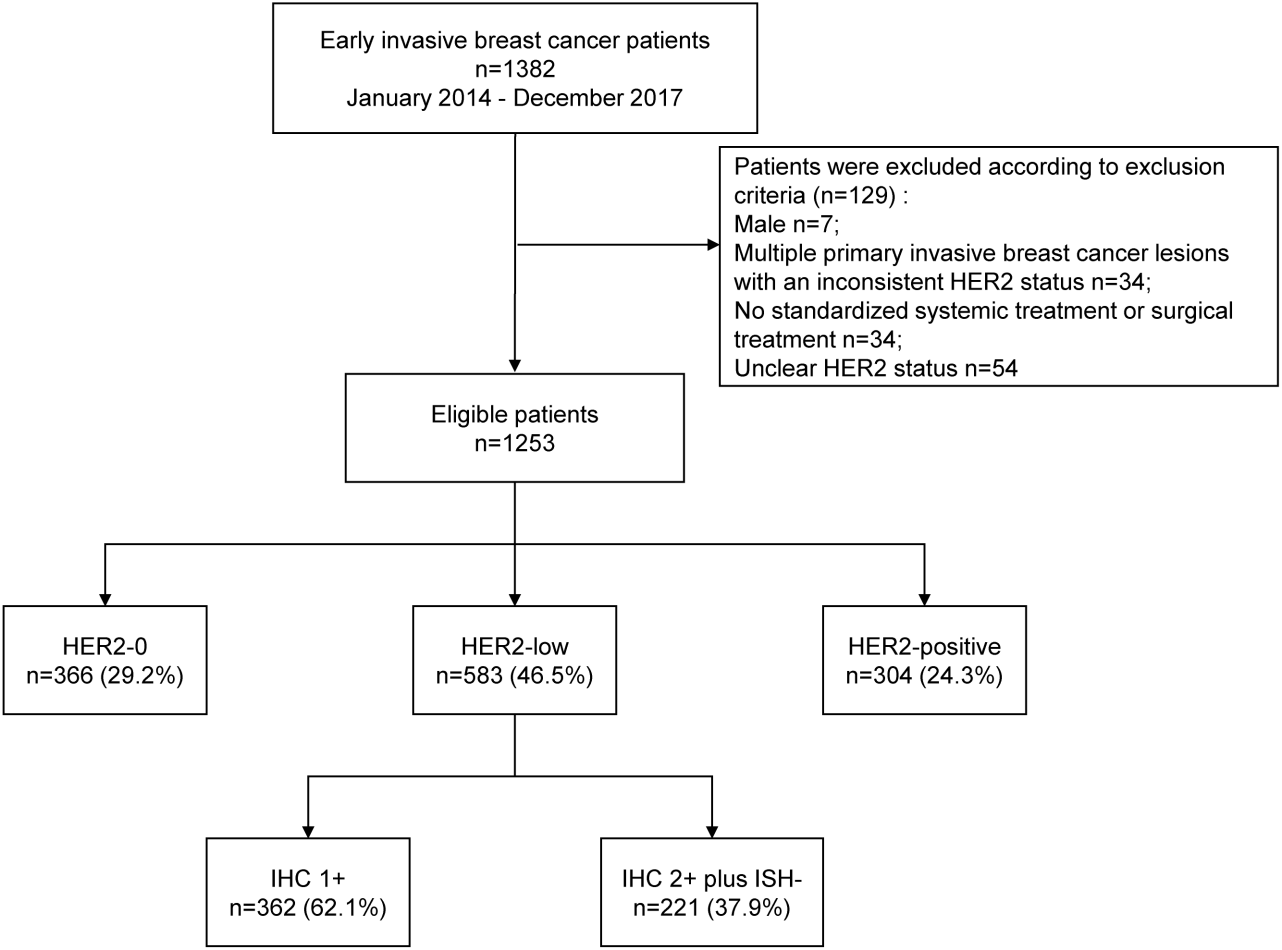
Flow diagram. IHC, immunohistochemistry; ISH, in situ hybridization.

From the database of the Breast Disease Center of Peking University First Hospital, a total of 1382 patients data with EBC were admitted from January 1, 2014, to December 31, 2017. After the exclusion of ineligible cases, 1253 cases of EBC were retrospectively analyzed including 583 HER2‐low cases, accounting for 46.5% of the sample; 366 HER2‐0 cases accounted for 29.2% of the sample (Figure 1). The median age of the 583 patients in the HER2‐low group was 55 years.

### 3.2 Baseline characteristics

All 583 HER2‐low BC patients were included in this study, 487 (83.5%) of whom were HR‐positive, while 96 (16.5%) were HR‐negative (Table 1). According to the anatomical staging of the AJCC (8th edition), among HER‐2 low patients, 220 (37.7%) were in stage I, 291 (49.9%) were in stage II, and 72 (12.3%) were in stage III (Table 1). Significant differences in N stage, histological type, histological grade, ER status, PR status, Ki67, basal‐like subtype, and axillary surgery method were identified between the low HER2‐low group and the HER2‐0 group (*p* < 0.05; Table 1). Among the HR‐positive patients, significant differences in age, N stage, histological subtype, tumor‐infiltrating lymphocytes (TILs), androgen receptor (AR) status, TOPIIa, and surgical modality were found between the HER2‐low and HER2‐0 BC patient groups (*p* < 0.05; Table 2). Among the HR‐negative patients, significant differences in age, menopause status, histological grade, Ki67, AR status, TOPIIa, and surgical modality were found between the HER2‐low and HER2‐0 BC patient groups (*p* < 0.05; Table 2). Further dividing HER2‐low into HER2 IHC 1+ and HER2 IHC 2+/ISH, IHC 1+ accounted for 62.1% of cases, and IHC2+/ISH− accounted for 37.9% of cases. Significant differences in T stage, histological subtype, histological grade, Ki67, TOPIIa, and surgical modality were found between the HER2 IHC 1+ and HER2 IHC 2+ plus ISH− BC patient groups (*p* < 0.05; Table 3).

**TABLE 1:** Baseline characteristics between the human epidermal growth factor receptor 2 (HER2)‐low and the HER2‐0 group.

	HER2‐0 (*n* = 366)	HER2‐low (*n* = 583)	*p* Value
Age			0.857
Median (IQR)	55 (46, 65)	55 (46, 64)	0.742^a^
<40	36 (9.8%)	54 (9.3%)	
≥40	330 (90.2%)	529 (90.7%)	
Menopausal status			
Postmenopausal	213 (58.2%)	328 (56.3%)	0.604
Premenopausal	153 (41.8%)	255 (43.7%)	
T stage			
T0‐1	200 (54.6%)	310 (53.2%)	0.739
T2	149 (40.7%)	250 (42.9%)	
T3‐4	17 (4.6%)	23 (3.9%)	
N stage			
N0	254 (69.4%)	342 (58.7%)	0.004
N1	83 (22.7%)	180 (30.9%)	
N2–3	29 (7.9%)	61 (10.5%)	
Anatomic stage			
I	161 (44.0%)	220 (37.7%)	0.130
II	169 (46.2%)	291 (49.9%)	
III	36 (9.8%)	72 (12.3%)	
Prognostic stage			
I	243 (67.7%)	410 (70.4%)	0.580
II	74 (20.6%)	115 (19.8%)	
III	42 (11.7%)	57 (9.8%)	
Unknown	7	1	
Histological type			
Ductal	296 (81.1%)	513 (88.0%)	0.014
Lobular	19 (5.2%)	19 (3.3%)	
Other	50 (13.7%)	51 (8.7%)	
Unknown	1	0	
Histological grade			
G1	82 (22.8%)	155 (26.6%)	0.021
G2	159 (44.3%)	284 (48.8%)	
G3	118 (32.9%)	143 (24.6%)	
Unknown	7	1	
Lymphovascular invasion and/or perineural invasion			
Yes	68 (18.6%)	121 (20.8%)	0.463
No	298 (81.4%)	462 (79.2%)	
ER status			
Positive	265 (72.4%)	487 (83.5%)	<0.001
Negative	101 (27.6%)	96 (16.5%)	
PR status			
Positive	232 (63.4%)	419 (71.9%)	0.006
Negative	134 (36.6%)	164 (28.1%)	
Ki67			0.265
Median (IQR)	25 (15, 58.75)	23 (15, 40)	0.011^a^
≤20%	123 (33.6%)	218 (37.4%)	
>20%	243 (66.4%)	365 (62.6%)	
TILs			
Median (IQR)	2 (1, 5)	2 (1, 5)	0.191^a^
Breast surgery			
Lumpectomy	133 (36.3%)	176 (30.2%)	0.058
Total mastectomy	233 (63.7%)	407 (69.8%)	
Axillary surgery			
SLNB	244 (66.7%)	333 (57.1%)	0.005^b^
ALND	120 (32.8%)	248 (42.5%)	
None	2 (0.5%)	2 (0.3%)	
Radiotherapy			
Yes	197 (54.9%)	295 (52.0%)	0.436
No	162 (45.1%)	272 (48.0%)	
Unknown	7	16	
Chemotherapy			
Neoadjuvant chemotherapy	53 (14.5%)	77 (13.2%)	0.712
Adjuvant chemotherapy	141 (38.5%)	239 (41.0%)	
None	172 (47.0%)	267 (45.8%)	
p53			0.649
Median (IQR)	0 (0, 20)	0 (0, 10)	0.820^a^
<1%	220 (60.6%)	338 (58.9%)	
≥1%	143 (39.4%)	236 (41.1%)	
Unknown	3	9	
PTEN			
0	61 (17.5%)	77 (13.5%)	0.134
1+	101 (28.9%)	162 (28.4%)	
2+	134 (38.4%)	258 (45.3%)	
3+	53 (15.2%)	73 (12.8%)	
Unknown	17	13	
CK5/6			
0	272 (76.6%)	496 (86.4%)	<0.001
1–3+	83 (23.4%)	78 (13.6%)	
Unknown	11	9	
Basal‐like			
Yes	86 (23.6%)	76 (13.3%)	<0.001
No	278 (76.4%)	497 (86.7%)	
Unknown	2	10	
AR			1.000
Median (IQR)	90 (30, 90)	80 (30, 90)	0.320^a^
0	68 (19.3%)	111 (19.4%)	
≥1%	284 (80.7%)	460 (80.6%)	
Unknown	14	12	
TOPIIa			0.668
Median (IQR)	15 (5, 30)	13 (5, 20)	0.309^a^
<5%	54 (15.3%)	80 (14.1%)	
≥5%	298 (84.7%)	488 (85.9%)	
Unknown	14	15	

Abbreviations: ALND, axillary lymph node dissection; AR, androgen receptor; ER, estrogen receptor; IQR, interquartile range; PR, progesterone receptor; SLNB, sentinel lymph node biopsy; TILs, tumor‐infiltrating lymphocytes.

^a^ Mann–Whitney test.

^b^ Fisher's exact test.

**TABLE 2:** Baseline characteristics by hormone receptor (HR) status.

	HR+			HR‐		
	HER2‐0 (*n* = 265)	HER2‐low (*n* = 487)	*p* Value	HER2‐0 (*n* = 101)	HER2‐low (*n* = 96)	*p* Value
Age			0.098			0.009
Median (IQR)	56 (47, 65)	54 (45, 64)	0.047^a^	52.72 ± 13.77	57.9 ± 11.78	0.005^c^
<40	16 (6%)	48 (9.9%)		20 (19.8%)	6 (6.2%)	
≥40	249 (94%)	439 (90.1%)		81 (80.2%)	90 (93.8%)	
Menopausal status						
Postmenopausal	162 (61.1%)	262 (53.8%)	0.063	51 (50.5%)	66 (68.8%)	0.014
Premenopausal	103 (38.9%)	225 (46.2%)		50 (49.5%)	30 (31.2%)	
T stage						
T0–1	163 (61.5%)	277 (56.9%)	0.468	37 (36.6%)	33 (34.4%)	0.469
T2	93 (35.1%)	191 (39.2%)		56 (55.4%)	59 (61.5%)	
T3–4	9 (3.4%)	19 (3.9%)		8 (7.9%)	4 (4.2%)	
N stage						
N0	184 (69.4%)	281 (57.7%)	0.005	70 (69.3%)	61 (63.5%)	0.220
N1	59 (22.3%)	159 (32.6%)		24 (23.8%)	21 (21.9%)	
N2–3	22 (8.3%)	47 (9.7%)		7 (6.9%)	14 (14.6%)	
Anatomic stage						
I	132 (49.8%)	197 (40.5%)	0.045	29 (28.7%)	23 (24%)	0.179
II	106 (40%)	235 (48.3%)		63 (62.4%)	56 (58.3%)	
III	27 (10.2%)	55 (11.3%)		9 (8.9%)	17 (17.7%)	
Prognostic stage						
I	215 (82.7%)	387 (79.6%)	0.537	28 (28.3%)	23 (24%)	0.789
II	30 (11.5%)	70 (14.4%)		44 (44.4%)	45 (46.9%)	
III	15 (5.8%)	29 (6%)		27 (27.3%)	28 (29.2%)	
Unknown	5	1		2	0	
Histological type						
Ductal	212 (80%)	430 (88.3%)	0.008	84 (84%)	83 (86.5%)	0.544
Lobular	19 (7.2%)	18 (3.7%)		0 (0%)	1 (1%)	
Other	34 (12.8%)	39 (8%)		16 (16%)	12 (12.5%)	
Unknown	0	0		1	0	
Histological grade						
G1	78 (30%)	146 (30%)	0.697	4 (4%)	9 (9.4%)	0.006
G2	140 (53.8%)	250 (51.4%)		19 (19.2%)	34 (35.4%)	
G3	42 (16.2%)	90 (18.5%)		76 (76.8%)	53 (55.2%)	
Unknown	5	1		2	0	
Lymphovascular invasion and/or perineural invasion						
Yes	55 (20.8%)	107 (22%)	0.768	13 (12.9%)	14 (14.6%)	0.887
No	210 (79.2%)	380 (78%)		88 (87.1%)	82 (85.4%)	
PR status						
Positive	232 (87.5%)	419 (86%)	0.640	–	–	
Negative	33 (12.5%)	68 (14%)		–	–	
Ki67			0.342			0.002
Median (IQR)	20 (10, 30)	20 (14, 30)	0.138^a^	70 (40, 85)	40 (20, 71.25)	<0.001^a^
≤20%	118 (44.5%)	198 (40.7%)		5 (5%)	20 (20.8%)	
>20%	147 (55.5%)	289 (59.3%)		96 (95%)	76 (79.2%)	
TILs						
Median (IQR)	1 (1, 5)	2 (1, 5)	0.040^a^	5 (1, 20)	5 (1, 20)	0.441^a^
Breast surgery						
Lumpectomy	96 (36.2%)	158 (32.4%)	0.333	37 (36.6%)	18 (18.8%)	0.008
Total mastectomy	169 (63.8%)	329 (67.6%)		64 (63.4%)	78 (81.2%)	
Axillary surgery						
SLNB	185 (69.8%)	279 (57.3%)	0.001^b^	59 (58.4%)	54 (56.2%)	0.828^b^
ALND	79 (29.8%)	206 (42.3%)		41 (40.6%)	42 (43.8%)	
None	1 (0.4%)	2 (0.4%)		1 (1%)	0 (0%)	
Radiotherapy						
Yes	139 (53.5%)	256 (53.8%)	0.995	58 (58.6%)	39 (42.9%)	0.043
No	121 (46.5%)	220 (46.2%)		41 (41.4%)	52 (57.1%)	
Unknown	5	11		2	5	
Chemotherapy						
Neoadjuvant chemotherapy	16 (6%)	44 (9%)	0.084	37 (36.6%)	33 (34.4%)	0.573
Adjuvant chemotherapy	96 (36.2%)	200 (41.1%)		45 (44.6%)	39 (40.6%)	
None	153 (57.7%)	243 (49.9%)		19 (18.8%)	24 (25%)	
p53			0.232			0.399
Median (IQR)	0 (0, 2)	0 (0, 4)	0.271^a^	80 (0, 90)	80 (0, 90)	0.884^a^
<1%	182 (68.9%)	308 (64.3%)		38 (38.4%)	30 (31.6%)	
≥1%	82 (31.1%)	171 (35.7%)		61 (61.6%)	65 (68.4%)	
Unknown	1	8		2	1	
PTEN						
0	31 (12%)	54 (11.3%)	0.665	30 (33%)	23 (24.5%)	0.370
1+	73 (28.3%)	130 (27.3%)		28 (30.8%)	32 (34%)	
2+	112 (43.4%)	227 (47.7%)		22 (24.2%)	31 (33%)	
3+	42 (16.3%)	65 (13.7%)		11 (12.1%)	8 (8.5%)	
Unknown	7	11		10	2	
CK5/6						
0	247 (96.9%)	468 (97.5%)	0.789	25 (25%)	28 (29.8%)	0.557
1–3+	8 (3.1%)	12 (2.5%)		75 (75%)	66 (70.2%)	
Unknown	10	7		1	2	
Basal‐like						
Yes	–	–		86 (86.9%)	76 (88.4%)	0.932
No	–	–		13 (13.1%)	10 (11.6%)	
Unknown				2	10	
AR			<0.001			<0.001
Median (IQR)	90 (80, 90)	90 (50, 90)	<0.001^a^	0 (0, 20)	57.5 (0, 90)	<0.001^a^
0	6 (2.3%)	75 (15.7%)		62 (66%)	36 (38.3%)	
≥1%	252 (97.7%)	402 (84.3%)		32 (34%)	58 (61.7%)	
Unknown	7	10		7	2	
TOPIIa			0.069			0.028
Median (IQR)	10 (5, 15)	10 (5, 20)	0.007^a^	30 (20, 60)	20 (10, 40)	<0.001^a^
<5%	51 (19.8%)	68 (14.3%)		3 (3.2%)	12 (13%)	
≥5%	207 (80.2%)	408 (85.7%)		91 (96.8%)	80 (87%)	
Unknown	7	11		7	4	

Abbreviations: ALND, axillary lymph node dissection; AR androgen receptor; ER, estrogen receptor; IQR, interquartile range; PR, progesterone receptor; SLNB, sentinel lymph node biopsy; TILs, tumor‐infiltrating lymphocytes.

^a^ Mann–Whitney test.

^b^ Fisher's exact test.

^c^ T test.

**TABLE 3:** Baseline characteristics between the epidermal growth factor receptor 2 (HER2) IHC 1+ and the HER2 IHC 2+ plus ISH− group.

	HER2 IHC 1+ (*n* = 362)	HER2 IHC 2+ plus ISH− (*n* = 221)	*p* Value
Age			0.562
Median (IQR)	55 (46, 64.75)	55 (46, 64)	0.835^a^
<40	36 (9.9%)	18 (8.1%)	
≥40	326 (90.1%)	203 (91.9%)	
Menopausal status			
Postmenopausal	198 (54.7%)	130 (58.8%)	0.374
Premenopausal	164 (45.3%)	91 (41.2%)	
T stage			
T0–1	208 (57.5%)	102 (46.2%)	0.030
T2	141 (39%)	109 (49.3%)	
T3–4	13 (3.6%)	10 (4.5%)	
N stage			
N0	218 (60.2%)	124 (56.1%)	0.611
N1	107 (29.6%)	73 (33%)	
N2–3	37 (10.2%)	24 (10.9%)	
Anatomic stage			
I	155 (42.8%)	65 (29.4%)	0.005
II	165 (45.6%)	126 (57%)	
III	42 (11.6%)	30 (13.6%)	
Prognostic stage			
I	267 (74%)	143 (64.7%)	0.009
II	57 (15.8%)	58 (26.2%)	
III	37 (10.2%)	20 (9%)	
Unknown	1	0	
Histological type			
Ductal	315 (87%)	198 (89.6%)	0.041
Lobular	17 (4.7%)	2 (0.9%)	
Other	30 (8.3%)	21 (9.5%)	
Histological grade			
G1	112 (31%)	43 (19.5%)	0.006
G2	170 (47.1%)	114 (51.6%)	
G3	79 (21.9%)	64 (29%)	
Unknown	1	0	
Lymphovascular invasion and/or perineural invasion			
Yes	75 (20.7%)	46 (20.8%)	1.000
No	287 (79.3%)	175 (79.2%)	
ER status			
Positive	305 (84.3%)	182 (82.4%)	0.627
Negative	57 (15.7%)	39 (17.6%)	
PR status			
Positive	263 (72.7%)	156 (70.6%)	0.658
Negative	99 (27.3%)	65 (29.4%)	
Ki67			<0.001
Median (IQR)	20 (10, 35)	30 (18, 40)	<0.001^a^
≤20%	159 (43.9%)	59 (26.7%)	
>20%	203 (56.1%)	162 (73.3%)	
TILs			
Median (IQR)	2 (1, 5)	2 (1, 5)	0.468^a^
Breast surgery			
Lumpectomy	122 (33.7%)	54 (24.4%)	0.023
Total mastectomy	240 (66.3%)	167 (75.6%)	
Axillary surgery			
SLNB	0 (0%)	2 (0.9%)	0.149^b^
ALND	212 (58.6%)	121 (54.8%)	
None	150 (41.4%)	98 (44.3%)	
Radiotherapy			
Yes	187 (53.1%)	108 (50.2%)	0.560
No	165 (46.9%)	107 (49.8%)	
Unknown	10	6	
Chemotherapy			
Neoadjuvant chemotherapy	44 (12.2%)	33 (14.9%)	<0.001
Adjuvant chemotherapy	128 (35.4%)	111 (50.2%)	
None	190 (52.5%)	77 (34.8%)	
p53			0.566
Median (IQR)	0 (0, 15)	0 (0, 10)	0.740^a^
<1%	214 (59.9%)	124 (57.1%)	
≥1%	143 (40.1%)	93 (42.9%)	
Unknown	5	4	
PTEN			
0	50 (14.1%)	27 (12.6%)	0.187
1+	92 (25.9%)	70 (32.6%)	
2+	161 (45.4%)	97 (45.1%)	
3+	52 (14.6%)	21 (9.8%)	
Unknown	7	6	
CK5/6			
0	305 (85.7%)	191 (87.6%)	0.594
1–3+	51 (14.3%)	27 (12.4%)	
Unknown	6	3	
Basal‐like			
Yes	45 (12.7%)	31 (14.2%)	0.688
No	310 (87.3%)	187 (85.8%)	
Unknown	7	3	
AR			0.683
Median (IQR)	80 (20, 90)	80 (30, 90)	0.927^a^
0	71 (20.1%)	40 (18.3%)	
≥1%	282 (79.9%)	178 (81.7%)	
Unknown	9	3	
TOPIIa			0.053
Median (IQR)	10 (5, 20)	15 (10, 25)	<0.001^a^
<5%	58 (16.4%)	22 (10.2%)	
≥5%	295 (83.6%)	193 (89.8%)	
Unknown	9	6	

Abbreviations: ALND, axillary lymph node dissection; AR androgen receptor; ER, estrogen receptor; IQR, interquartile range; PR, progesterone receptor; SLNB, sentinel lymph node biopsy; TILs, tumor‐infiltrating lymphocytes.

^a^ Mann–Whitney test.

^b^ Fisher's exact test.

### 3.3 Prognostic analysis

All enrolled patients underwent the follow‐up, with a median follow‐up time of 53 months (95% confidence interval (CI): 50.9–55.1).

A total of 46 HER2‐low patients (7.9%) had events, and the 5‐year DFS was 90.2% (95% CI: 87.2–93.1). Twenty‐three patients in this group died (3.9%), for a 5‐year OS of 95.4% (95% CI: 93.3–97.6). Among them, the 5‐year DFS of the HR‐positive patients was 92.2% (95% CI: 88.1–94.2), and their 5‐year OS was 95.8% (95% CI: 93.5–98.1); the 5‐year DFS of the HR‐negative patients was 85.4% (95% CI: 76.7–94.0), and their 5‐year OS was 93.4% (95% CI: 87.7–99.2).

**FIGURE 2:**
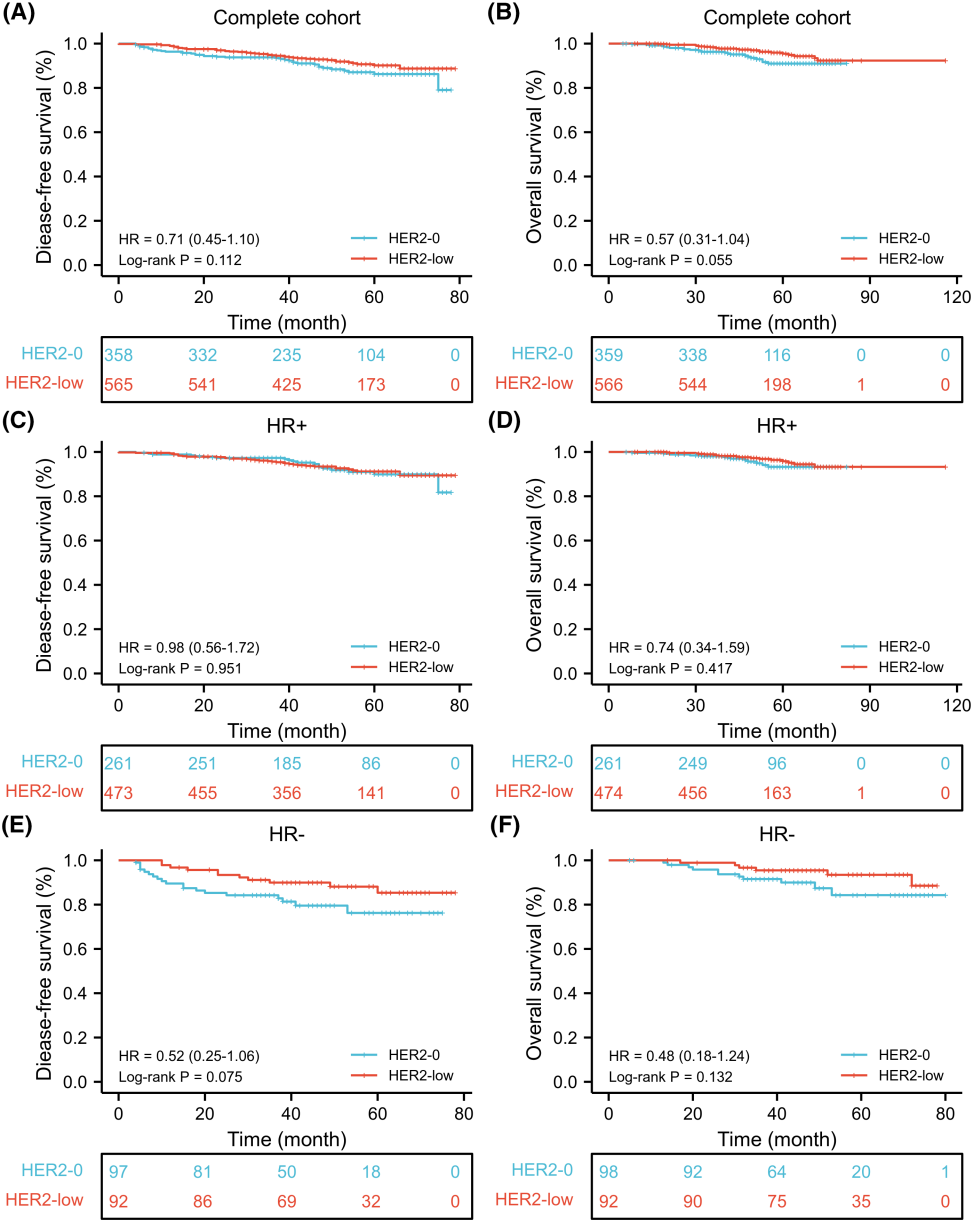
Comparison of HER2‐low and HER2‐0 breast cancer for disease‐free survival and overall survival. (A, B) complete cohort. (C, D) HR‐positive tumors. (E, F) HR‐negative tumors. HR, hormone receptor.

No significant difference in DFS or OS was found between the HER2‐low and HER2‐0 groups of patients (DFS: log‐rank *p* = 0.112; OS: log‐rank *p* = 0.055; Figure 2A,B). In HR‐positive patients, the DFS and OS of HER2‐low patients were similar to HER2‐0 patients (DFS: log‐rank *p* = 0.951; OS: log‐rank *p* = 0.417; Figure 2C,D). Similar results were yielded in the HR‐negative patients between the HER2‐low and HER2‐0 group (DFS: log‐rank *p* = 0.075; OS: log‐rank *p* = 0.132; Figure 2E,F), but a tendency of better prognosis of HER2‐low group was seen in HR‐negative tumors.

Univariate and multivariate Cox regression analyses of the prognostic factors of patients with low HER2 expression showed that T stage, lymphovascular invasion, and/or perineural invasion were prognostic factors for HER2‐low patients (Table 4).

**TABLE 4:** Univariate and multivariate Cox regression analysis of prognostic factors for disease‐free survival in the epidermal growth factor receptor 2 (HER2)‐low group.

	Univariate analysis			Multivariate analysis		
	HR	95%CI	*p* Value	HR	95%CI	*p* Value
HER2 status						
IHC 1+	Ref.					
IHC 2+ plus ISH−	1.31	0.73–2.35	0.363			
Age						
<40	Ref.					
≥40	0.53	0.24–1.19	0.125			
Menopausal status						
Postmenopausal	Ref.					
Premenopausal	0.64	0.25–1.18	0.150			
T stage						
T0–1	Ref.			Ref.		
T2–4	2.51	1.35–4.64	0.003	2.38	1.44–3.95	0.001
N stage						
N0	Ref.			Ref.		
N1–3	1.78	1.00–3.18	0.051	0.85	0.44–1.67	0.645
Histological grade						
G1	Ref.			Ref.		
G2‐3	4.16	1.49–11.61	0.006	2.38	0.80–7.11	0.119
Lymphovascular invasion and/or perineural invasion						
No	Ref.			Ref.		
Yes	3.00	1.67–5.41	<0.001	3.04	1.60–5.77	0.001
Ki67						
<30%	Ref.			Ref.		
≥30%	2.42	1.34–4.37	0.004	1.83	0.92–3.63	0.083
HR status						
Positive	Ref.					
Negative	1.63	0.83–3.22	0.156			
Chemotherapy						
No	Ref.			Ref.		
Yes	1.97	1.05–3.69	0.034	0.99	0.49–2.01	0.972

Abbreviation: HR, hormone receptor.

## 4 DISCUSSION

Breast cancer is currently the malignant tumor with the highest incidence among women. In 2020, 2.26 million new cases of BC were reported worldwide, and 420,000 new cases were reported in China.^15^ Since the beginning of the 21st century, as our understanding has improved, BC has gradually entered the era of classified treatment. Different molecular subtypes are not only closely related to prognosis but also become the basis for making clinical treatment decisions. The NCCN and CSCO proposed focusing on BC with low HER2 expression in 2021.^2^, ^3^ Therefore, we conducted studies on HER2‐low EBC patients treated at the Breast Disease Center of Peking University First Hospital and analyzed their clinicopathological characteristics and prognostic factors.

HER2 amplification and/or overexpression in breast cancer has been identified as a significant driver of tumorigenesis, serving as a crucial prognostic biomarker and therapeutic target. The targeting of HER2 has demonstrated substantial enhancements in the prognosis of patients diagnosed with HER2‐positive breast cancer.^21^ Consequently, for over two decades, HER2 has been dichotomously classified as positive or negative.^7^ Regrettably, HER2‐low expression has been overlooked as lacking clinical implications, thus categorized as HER2‐negative. However, the emergence of innovative anti‐HER2 ADCs has revealed through the findings of the DESTINY‐Breast04 trial that individuals with HER2‐low BC can also derive advantages from DS‐8201a treatment.^22^ This expansion of indications and the beneficiary population for anti‐HER2 therapy signifies a significant development. Breast cancer continues to be the most prevalent form of cancer, with an estimated 300,000 new cases projected to be diagnosed in the United States in 2022.^23^ Among these cases, the subgroup of HER2‐low tumors constitutes a substantial proportion, ranging from 45% to 55% of the overall breast cancer population, thereby warranting significant attention and consideration.^16^ Hence, the academic community has initiated the reclassification of HER2 expression levels into three distinct categories, namely HER2‐positive, HER2‐low, and HER2‐0.^24^ The introduction of the newly proposed HER2‐low category holds significant implications as it may serve as a novel prognostic predictor or a screening indicator for treatment efficacy in the population. This development presents a noteworthy advancement and poses a challenge to the existing categorization and therapeutic approaches in BC. However, the limited attention given to HER2‐low BC has resulted in a dearth of knowledge regarding its characteristics and behavior.

Fortunately, notable discoveries have emerged in this particular field. A comprehensive analysis of a retrospective cohort on a substantial scale, encompassing 392,246 cases of HER2‐0 BC and 743,770 cases of HER2‐low BC sourced from the National Cancer Database, found minimal prognostic differences between HER2‐low and HER2‐0 breast cancer.^25^ Significantly, the presence of ER expression has emerged as a potential confounding factor in prognostic analyses within this context, as HER2‐low tumors tend to be predominantly composed of highly ER‐expressing tumors, while ER‐low tumors are primarily observed among HER2‐0 tumors.^26^ In a similar vein, the application of PAM50 analyses was undertaken to ascertain potential disparities in gene expression between HER2‐low and HER2‐0 tumors. In HER2‐low tumors, there is a higher prevalence of luminal A and luminal B subtypes compared to HER2‐0 tumors, while the occurrence of basal‐like subtype is lower than that observed in HER2‐0 tumors, thus aligning with the expression of ER.^19^ However, a meta‐analysis conducted on a cohort of 636,535 patients across 23 studies revealed that the HER2‐0 subgroup exhibited a higher prevalence of unfavorable risk factors.^27^ Stratification based on HR status demonstrated a correlation between premenopausal status and HER2‐0 tumors in the HR‐positive subgroup, while young age, grade 3 tumors, and advanced T stage were associated with HER2‐0 tumors in the HR‐negative subgroup. Additionally, irrespective of HR subtype, HER2‐low status exhibited a positive association with improved DFS and OS outcomes across all patients. Currently, a conclusive determination regarding the clinicopathologic features, prognosis, and biological characteristics of HER2‐low BC has not yet been reached.

In this study, we found that among HER2‐low BC patients, HR‐positive BC accounted for 83.5% of cases, which is similar with the reported data.^18^, ^19^, ^20^ Therefore, the hypothesis that ER pathway and HER2 pathway may be related to each other is logical. Crosstalk between HER2 and ER pathways is a potential mechanism of tumor adaptation and drug resistance to endocrine therapy, mainly tamoxifen.^28^, ^29^ But we found no significance in DFS or OS between HER2‐low and HER2‐0 HR‐positive patients. If anti‐HER2 therapy is applied in HER2‐low patients in the future, the combination with endocrine therapy may be synergistic.

Another interesting result is the AR expression diversity with HR expression in HER2‐low and HER2‐0 group. In HR‐positive patients, AR‐positive percentage was higher in HER2‐0 group. However, in HR‐negative patients, AR‐positive percentage was higher in HER2‐low group. It is suggested that in LAR subtype of TNBC, ERBB2 mutation frequency is higher than in other subtypes.^30^ In addition, the prognostic value of AR for patients remains uncertain, which can be opposite in different subtypes.^31^ We would like to make a venture guess, there is a potential complex crosstalk among ER, AR, and HER2 pathways, but further evidences are needed to confirm it. And based on the further investigation, maybe AR‐targeted therapy could play a role in selected patients.

In addition, TOPIIa expression is different in HER2‐low patients. The expression level of TOPIIa in patients with HER2 IHC 2+ plus ISH− is higher than HER2 IHC 1+. However, when compare HER2 low with HER2‐0, the TOPIIa expression is higher in HER2‐0 than HER2‐low HR‐negative patients. TOPIIa is a target for doxorubicin and is coamplified in 20%–50% of HER2‐amplified tumors. But with current evidence, neither HER‐2 nor TOPIIa gene status can be considered clinically valuable markers for anthracycline benefit.^32^

Since HER2‐low BC has begun to receive attention, relevant studies have investigated the difference between HER2‐low BC and HER2‐0 BC. Most of the results show that the prognosis of HER2‐low BC was not significantly different from that of HER2‐0 BC.^18^, ^19^, ^20^, ^33^, ^34^, ^35^ One study found that the prognosis of HER2‐low BC is better than that of HER‐0 BC.^36^ However, another study found that HER2‐low patients had a worse prognosis than HER2‐0 patients among HR‐positive BC patients.^17^ In this study, we compared HER2‐low BC to HER2‐0 BC and found no significant difference in prognosis between them, which is consistent with the results of most of the above studies. But in HR‐negative tumors, a tendency of better prognosis was seen in HER2‐low versus HER2‐0, which is consistent with the result of a Japanese study.^18^

The classification of HER2‐low status as an independent entity in breast cancer and its prognostic significance remain a topic of debate. The majority of previous studies have not identified significant variations in survival rates associated with HER2‐low status, suggesting that any potential prognostic relationship with HER2‐low status is likely to be nuanced. In our research, we observed a propensity toward improved prognosis in HER2‐low compared to HER2‐0 among HR‐negative tumors. It is plausible that HER2‐low BC is linked to the LAR subtype of TNBC, as evidenced by the higher prevalence of AR positivity in HER2‐low BC and the elevated occurrence of the HER2‐enriched subtype according to the PAM50 classification system in patients with LAR TNBC.^37^ Further investigation is warranted to assess the extent to which the prognostic correlation of HER2‐low TNBC can be attributed to the enrichment of the LAR subtype, given the improved prognosis yet diminished response to chemotherapy observed in AR‐positive TNBC.^38^

The prognostic implications of HER2‐low BC will be defined by the introduction of ADCs, considering the marginal nature of the observed survival differences.

The presence of inaccuracies in IHC may have a detrimental impact on the accurate determination of survival disparities linked to HER2‐low expression and could potentially be a contributing factor to the inconsistent prognostic associations observed in existing literature. Enhancing the precision of quantifying HER2 expression levels could potentially facilitate the evaluation of associations with patient outcomes. A fact of utilizing IHC for HER2 in the classification of these tumors is that the primary purpose of this assay is not to differentiate between HER2‐low and HER2‐0 tumors, but rather to distinguish HER2‐positive tumors that exhibit response to conventional monoclonal antibodies like trastuzumab. It is evident that there exists an urgent requirement to accurately quantify minimal levels of HER2 expression in order to identify patients who may derive benefits from potent ADCs, as demonstrated in the DAISY trial where even patients classified as HER2‐0 displayed response to DS‐8201a.^39^

The lack of identifiable prognostic and biological factors should not be interpreted as an indication that low HER2 expression lacks clinical significance. Conversely, the findings from the DESTINY‐Breast04 trial have established this biomarker as a crucial determinant in clinical decision‐making. The efficacy of DS‐8201a is not reliant on the characteristics of HER2‐low BC, but rather on the distinctive mechanism exhibited by ADCs. The most recent generation of ADCs, known as DS‐8201a, comprises an antibody, a tetrapeptide‐based cleavable linker, and a topoisomerase I inhibitor payload. The linker exhibits selectivity in its cleavage by cathepsins, which are up‐regulated in cancer cells, thereby liberating the payload.^40^ This payload, possessing membrane permeability, facilitates cytotoxicity toward neighboring cancer cells through a phenomenon referred to as the bystander effect.^40^ Hence, DS‐8201a demonstrates remarkable efficacy even in tumors characterized by low HER‐2 expression status.^22^ Further advancements may arise as a result of ongoing trials, such as the phase III, randomized DESTINY‐Breast06 trial, which aims to incorporate a group of patients exhibiting an ultralow HER2 score (HER2 IHC > 0 < 1+ expression). This trial has the potential to broaden the population that can benefit from DS‐8201a. In current clinical practice, HER2‐low and HER‐0 patients are both classified as HER2‐negative, with no difference in treatment regimens or prognosis as described above. A clinical trial of DS‐8201a, a novel ADC, showed that HER2‐low BC can benefit from DS‐8201a treatment, providing a novel treatment option for HER2‐low patients.^4^ A phase II clinical trial (ClinicalTrials.gov identifier: 04553770) is also being conducted in the neoadjuvant phase among HR‐positive HER2‐low EBC patients. After clinical trial verification, the prognosis of HER2‐low EBC patients should be further improved by receiving DS‐8201a treatment in the future.

This study was a single‐center retrospective study with a relatively small number of cases and a relatively short follow‐up time. To further explore the characteristics and prognosis of HER2‐low BC, larger‐scale, scientifically designed prospective studies are necessary to draw more rigorous conclusions.

Novel ADCs, such as DS‐8201a, provide good prospects for expanding the indications for anti‐HER2 treatment and the population that may gain a prognostic benefit. At the same time, clinical needs for HER2 status evaluation from qualitative diagnosis to expression level quantification have been proposed, which may serve as novel prognostic predictors or screening indicators for populations benefiting from treatment. These represent important advances and challenges to the concept of individualized BC treatment.

## 5 CONCLUSIONS

Our study indicated that HER2‐low EBC is a relatively large population, accounted for 46.5% of the EBC patient population. The tumor burden and invasiveness are prognostic factors of HER2‐low EBC. No statistically significant difference in prognosis was observed between HER2‐low and HER2‐0 BC patients with existing treatment. But a tendency of better prognosis was seen in HER2‐low patients versus HER2‐0, especially in HR‐negative tumors.

## AUTHOR CONTRIBUTIONS

**Qian Wu:** Conceptualization (lead); data curation (equal); formal analysis (lead); investigation (equal); software (lead); supervision (equal); validation (equal); visualization (equal); writing – original draft (lead); writing – review and editing (equal). **Fan Yang:** Conceptualization (supporting); data curation (equal); investigation (supporting); methodology (supporting); supervision (supporting); writing – original draft (equal); writing – review and editing (equal). **Yinhua Liu:** Investigation (supporting); project administration (supporting); resources (supporting); validation (supporting). **Hong Zhang:** Conceptualization (supporting); investigation (supporting); software (equal); validation (equal). **Shuang Zhang:** Investigation (supporting); software (supporting); supervision (supporting); validation (equal). **Ling Xin:** Conceptualization (supporting); data curation (supporting); investigation (supporting); supervision (supporting). **Ling Xu:** Conceptualization (lead); funding acquisition (equal); project administration (lead); supervision (lead); validation (supporting); writing – review and editing (supporting).

## FUNDING INFORMATION

This work was funded by National Key R&D Program of China (No. 2016YFC0901302), Interdisciplinary Clinical Research Project of Peking University First Hospital (2019CR38), and Beijing Medical Award Foundation (YXJL‐2020‐0941‐0736).

## CONFLICT OF INTEREST STATEMENT

The authors declare that they have no conflict of interest.

## ETHICS STATEMENT

This retrospective study involving human participants was performed in accordance with the ethical standards of the institutional and national research committee and with the 1964 Helsinki Declaration and its later amendments or comparable ethical standards. The study was approved by the Biomedical Research Ethics Committee of Peking University First Hospital (No. 2021 Research 206).

## CONSENT

Informed consent was waived due to the retrospective nature of the study.

## Supporting information


Figure S1.

## Data Availability

The datasets generated during and/or analyzed during the current study are available from the corresponding author Ling Xu on reasonable request.
