# Supplementary figures and images for: Analysis of clinicopathological characteristics and prognostic factors of early‐stage human epidermal growth factor receptor 2 (HER2)‐low breast cancer: Compared with HER2‐0 breast cancer
Source: Cancer Med. 2023 Sep 29;12(19):19560–75. doi: 10.1002/cam4.6571 (PMC10587975; doi:10.1002/cam4.6571)

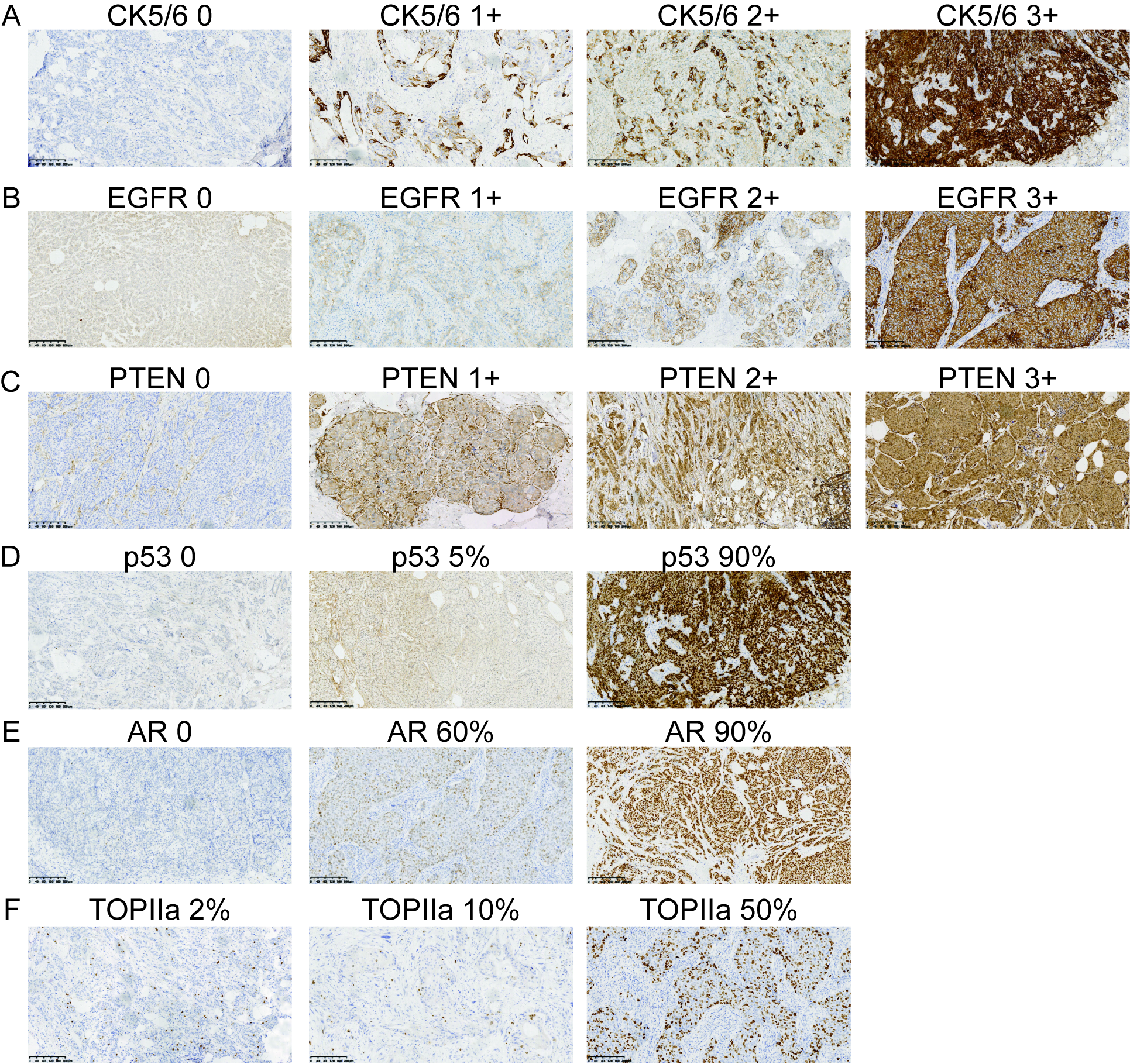

Supplement: Supplementary file 1 — Figure S1. [file CAM4-12-19560-s001.tif]
